# Milk Osteopontin and Human Health

**DOI:** 10.3390/nu15112423

**Published:** 2023-05-23

**Authors:** Esben S. Sørensen, Brian Christensen

**Affiliations:** Department of Molecular Biology and Genetics, Aarhus University, DK-8000 Aarhus, Denmark; bc@mbg.au.dk

**Keywords:** osteopontin, milk, bioactivity, gastrointestinal digestion, infant health, immune regulation, microbiota, intestinal development

## Abstract

Osteopontin (OPN) is a multifunctional protein found in all vertebrates. OPN is expressed in many different cell types, and is consequently found in most tissues and physiological secretions. OPN is involved in a multitude of biological processes, such as activation and regulation of the immune system; biomineralization; tissue-transformative processes, including growth and development of the gut and brain; interaction with bacteria; and many more. OPN is found in the highest concentrations in milk, where it is believed to initiate and regulate developmental, immunological and physiological processes in infants who consume milk. Processes for the isolation of bovine OPN for use in infant formula have been developed, and in recent years, many studies have investigated the effects of the intake of milk OPN. The purpose of this article is to review and compare existing knowledge about the structure and function of milk OPN, with a particular focus on the effects of milk OPN on human health and disease.

## 1. Introduction

Osteopontin (OPN) is a pleiotropic protein found in tissues and body fluids throughout the animal kingdom. OPN was originally described as a secreted phosphoprotein related to transformation [1], and it was later identified in the extracellular matrix of bovine bone [2]. The name osteopontin (“osteo” is Greek for bone and “pontin” is derived from the Latin word “pons”, meaning bridge) was proposed by Oldberg et al. due to its ability to function as a bridge between cells and the mineralized phase of bone [3]. OPN is also known as early T-lymphocyte activation gene 1 (Eta-1) [4], bone sialoprotein I (BSP I) [2], secreted phosphoprotein-1 (SPP-1) [5], uropontin [6] and lactopontin [7]. This variety of names reflects the multifunctionality of OPN and the diversity of processes in which it is involved.

OPN is encoded by a single gene, but is expressed by many different cell types and subjected to alternative splicing and extensive post-translational modification, depending on the site of expression. OPN is involved in numerous processes including, but not limited to, biomineralization, the regulation of immune cell function, cancer metastasis, and development of the mammary gland, brain, and intestines [8,9].

In 1989, OPN was purified from human milk, both as an intact protein and as fragments presumably generated by proteolytic cleavage in milk [10]. A few years later, OPN was isolated and characterized from bovine milk [11]. Recently, the bovine milk protein ingredient Lacprodan OPN-10© (Arla Foods Ingredients), consisting of +95% bovine OPN, has been recommended for use as Novel Food in infant formula by The European Food Safety Authorities (EFSA) [12]. Milk OPN has previously been reviewed by several groups [13,14,15,16,17,18]. In the present review, we summarize existing knowledge about human and bovine milk OPN, with emphasis on the cellular and physiological effects of milk OPN as a dietary ingredient.

## 2. Osteopontin in Milk

Milk is evolutionarily optimized to deliver offspring essential nutrients to be used as building blocks and energy. On top of this, milk contains many bioactive molecules, such as lipids, carbohydrates and proteins, that have an impact on infant development and health. Many of these bioactive components are proteins that, either directly or via encrypted peptides released during digestion, mediate functionalities such as antimicrobial activity, immunomodulation, mineral-binding and the regulation of blood pressure [19]. Well-known examples are lactoferrin, which is an important carrier of iron, and furthermore, releases an antibacterial fragment upon digestion [20]. Caseins, especially β-casein, are susceptible to proteolytic cleavage in milk, presumably due to their lack of secondary structure [21]. Several bioactivities, such as mineral-binding, immune modulation, and anti-oxidative and antimicrobial effects, are associated with casein peptides [22]. α-lactalbumin and β-lactoglobulin encrypt, in their sequences, several peptides with biological effects, such as antihypertensive, opioid, antitumor antioxidative and immunomodulation [23,24,25]. In addition, many relatively minor milk proteins, such as haptocorrin, lysozyme, EPV20 and MFGM proteins, have been shown to possess biological functions that may be beneficial for the offspring or the consumer of the milk [14]. Among these so-called minor milk proteins, osteopontin (OPN) has gained a lot of attention in recent years. OPN has been thoroughly characterized in bovine and human milk, but has also been identified and quantitated in the milk of several other mammalian species.

In orangutans (*Pongo pygmaeus*), OPN is present in a relatively high concentration in early milk (determined by mass spectrometric analysis; no absolute quantification has been reported) before decreasing at day 177 through the remainder of the first year of lactation [26]. In gorillas (*Gorilla gorilla*), OPN showed a decrease to a minimum at 242 days, before increasing and stabilizing for the rest of the first year of lactation [26]. A study using UHPLC-MS/MS (ultra-high-performance liquid chromatography tandem mass spectrometry) estimated the OPN concentrations in milk from cow (*Bos taurus*), buffalos (*Bison bison*), sheep (*Ovis aries*), goats (*Capra hircus*), and yaks (*Bos grunniens*). Two measurements were reported for all species: cows (51.4 mg/L and 56.4 mg/L), buffalos (68.5 mg/L and 51.8 mg/L), yaks (78.6 mg/L and 76.8 mg/L), sheep (41.06 mg/L and 29.8 mg/L), and goats (44.3 mg/L and 12.7 mg/L) [27]. A comprehensive proteomic study of camel milk showed great seasonal changes in the concentration of OPN, with a 50% increase in OPN levels from winter to summer [28]. In mouse milk, the concentration of OPN was measured by ELISA, showing mean levels of 150 mg/L in early lactation (day 0–3) that decreased to ~10 mg/L after day 8 [29].

From a human health and commercial perspective, human and bovine OPNs have attracted the most attention, and this review will focus on milk OPN from these species.

### 2.1. Osteopontin in Bovine Milk

OPN was first identified and isolated from proteose-peptone (the heat and acid stable fraction of cow’s milk) in 1993 [11]. The purification method included heating of the milk to 90 °C, followed by an adjustment of pH to 4.6 to precipitate caseins and the majority of the denatured whey proteins. OPN is an intrinsically disordered protein and will remain in solution even after these relatively harsh heat and acid treatments. After this, it was purified using a combination of size-exclusion and ion-exchange chromatography. Since then, more gentle methods for OPN purification have been developed; 8 mg OPN was isolated from 1 L of bovine milk using anion-exchange followed by hydrophobic-interaction chromatography [30], and 11 mg OPN was purified from 1 L bovine milk in a two-step procedure based on anion exchange [31]. Presently, OPN is purified to +95% purity for use in infant formula in large-scale dairy operation based on anion exchange [32,33,34].

The level of OPN in bovine milk has been estimated in several studies. A comprehensive study comprising milk from 661 Danish Holstein cows showed an average OPN concentration of 23.0 mg/L [35]. This concentration is in line with the average concentration of 18 mg/L determined for pooled dairy milk [36]. In a study analyzing the OPN content in milk from only five cows, OPN levels were found to be significantly higher, at around ~70 mg/L from parturition to lactation week 25, after which the concentration increased to ~150–250 mg/L between weeks 25 and 30 [37]. However, the cows in this study were selected based on specific genetic variations that influenced OPN promotor activity [37]. In a study using the MS/MS method on two bovine milk samples, the OPN concentration was estimated to be 53.9 mg/L [27].

Christensen et al. (2021) showed considerable individual variation in OPN milk content between Danish Holstein cows, with levels varying from 0.4 mg/L to 67.8 mg/L. In addition, it was shown that OPN levels decreased with parity and that the OPN content increased significantly with days in milk [35]. The latter agrees with the study by Dudemaine et al. (2014), where a significant increase in bovine OPN levels was observed after lactation week 25 [37]. It has been suggested that OPN is associated with lactation persistency and is involved in tissue remodeling [38]. The changes in OPN levels in milk could be a response to changes in the mammary gland or changes in milk protein composition as a function of days in milk, which has been reported in more studies [39,40].

The OPN gene has been associated with milk production traits and lactation persistency [38,39,40]. Interestingly, the OPN concentration in milk from Danish Holstein cows is a heritable parameter, but the proportion of the total phenotypic variance explained by the herd has been found to be low, demonstrating that management factors such as feed regime have very little impact on the OPN concentration in bovine milk [35].

### 2.2. Osteopontin in Human Milk

OPN in human breast milk was first described in 1989 by Senger et al. [10]. In 2004, Nagatomo et al. reported human breast milk OPN concentrations of 1493.4 mg/L at 3–7 days postpartum and 896.3 mg/L after one month of lactation [41]. This concentration would indicate that OPN comprises around 10% of all protein in human milk. This finding was questioned a few years later, as the ELISA method (IBL, Gunma, Japan) used is not validated for measurements in milk [36]. Schack et al. (2009) used an in-house-developed ELISA based on polyclonal antibodies raised against purified human milk OPN to analyze the OPN concentration in milk from 29 women (mean postpartum 20.4 days), and reported an average OPN concentration of 138 mg/L in breast milk, constituting, on average, 2.1% of the protein in human milk [36]. In a large multicenter cohort study, the milk OPN levels in 829 milk samples from 629 mothers from China, Japan, South Korea and Denmark were determined using a Quantikine Human Osteopontin ELISA (R&D Systems, Minneapolis, MN, USA) [42]. A large geographic difference in mean OPN levels was observed, ranging from 99.7 mg/L in Danish (average of 17.4 weeks postpartum) to 185.0 mg/L in Japanese (average of 9.1 weeks postpartum), 216.2 mg/L in Korean (average of 3.9 weeks postpartum) and 266.2 mg/L in Chinese mothers (average of 3.9 weeks postpartum) [42]. These levels are in accordance with recent studies that, via ELISA and UPLC-MS/MS, have determined milk OPN levels in Chinese mothers to be 300–350 mg/L (1–2 weeks postpartum) and 137.1 mg/L (third month of lactation), [43,44,45] and in Turkish and American mothers to be 178 mg/L (1 week postpartum) [46,47]. The levels of OPN in breast milk from the various studies are summarized in Table 1.

The reason for the geographical differences in milk OPN levels is not clear, but it seems that the concentration of OPN decreases with infant age, which hampers the comparison of the determined levels [42,43,45,47,48]. For instance, OPN in milk from 12 mothers from California was measured during their first year of lactation, showing high levels in the first week (178.00 mg/L) followed by a gradual decrease in the second week (137.8 mg/mL), after which the levels further decreased to 65 mg/mL after 1 month and remained at approximately 50 mg/L for up to 12 months [47]. Likewise, OPN levels of more than 300 mg/L (1–14 days postpartum) and 100–200 mg/mL (between 4–7 months postpartum) have been reported in Chinese milk samples [43,45].

**Table 1:** Human Milk Osteopontin Levels.

Study	Postpartum Period	# Women	OPN (mg/L)	Method	Country	Comments
[41]	72 h–7 days	23	1493.4	IBL ELISA	Japan	10% of milk protein is OPN
	1 month	26	896.3			
	4–7 months	20	550.8			
	11–14 months	15	412.7			
[49]	6–58 days	29	138	R&D ELISA	Denmark	2.1% of milk protein is OPN
[42]	4.3 weeks	76	266.2	R&D ELISA	China	2.7% of milk protein is OPN
	7.4 weeks	318	99.7		Denmark	1.3% of milk protein is OPN
	9.1 weeks	118	185.0		Japan	2.4% of milk protein is OPN
	3.9 weeks	117	216.2		Korea	1.8% of milk protein is OPN
[47]	1–7 days	12	178.0	R&D ELISA	USA	
	8–14 days		134.8			
	1 month		65.8			
	4 months		48.8			
	5 months		55.9			
	12 months		48.3			
[50]	Colostrum	33	180	MS/MS	USA	OPN level increases from colostrum to 2 weeks postpartum, and then, decreases
	2 weeks		330			
	5 weeks		300			
	10 weeks		260			
	13 weeks		190			
	17 weeks		200			
	24 weeks		150			
[46]	3 months	85	137.1	R&D ELISA	Turkey	OPN associated with birth route, energy intake, obesity and smoking but not associated with maternal age.
[48]	1–5 days	51	718	UHPLC-MS/MS	China	Maternal age and education contribute to OPN levels at 6 months. Mode of delivery and BMI show no effect.
	8–14 days		586			
	1 month		450			
	6 months		236			
[45]	7 days	38	334.71	UHPLC-MS/MS	China	Higher maternal age and BMI are associated with higher OPN levels. Mode of delivery shows no effect.
	14 days	48	264.76			
	28 days	43	210.45			
	120 days	36	112.10			
[43]	1–14 days	106	343.2	R&D ELISA	China	OPN levels are positively correlated with BMI, body weight and skeletal muscle.
	2–4 months		228.2			
	5–7 months		204.8			
[51]	Not specified	Pooled milk	>300	ELISA Invitrogen	USA	
[52]	1–7 days 20–38 days	48	318.1 ^1^	R&D ELISA	Japan	Higher OPN levels in colostrum after C-section. OPN levels in mature milk do not correlate with birth route.
		49	137.9 ^2^			
		48	300.8 ^1^			
		49	280.9 ^2^			

^1^ Milk from 1989, ^2^ milk from 2013.

Downward longitudinal changes in human milk OPN have been observed in term and preterm milk; however, there have been indications of higher OPN levels in transitional and early mature preterm milk compared to term milk [45]. In contrast to this trend, a single study on 31 American mothers reported higher OPN levels in milk 2–10 weeks postpartum compared to colostrum [50]. Thus, the difference in OPN levels reported between geographical sites [42] could partly be a consequence of the milk being collected at different times postpartum. Furthermore, the existence of geographical differences is questioned by studies reporting OPN levels of ~300–350 mg/L in milk from American mothers at undefined days postpartum [51] and 2–5 weeks postpartum [50]. These levels are comparable to the levels reported for Chinese mothers [42].

Maternal factors influencing the level of OPN in breast milk have been investigated by correlating OPN levels with the health status and dietary patterns of 85 Turkish mothers [46]. The study suggested that smoking, energy and fiber intake, and weight gain/high BMI during lactation all are associated with lower OPN levels in milk, whereas maternal age had no effect on the levels. Contradicting this, other studies have shown a positive correlation between milk OPN levels and body weight and BMI in lactating women [43,45], and that higher maternal age and education levels were correlated with higher OPN levels six months postpartum [48]. Interestingly, women who delivered vaginally had higher OPN levels in their milk compared to women who gave birth via C-section [46]. This could be explained by oxytocin expression during vaginal birth, as oxytocin induces the expression of OPN [53]. However, other studies report no differences in OPN levels in milk from mothers who delivered vaginally compared to those who delivered via a C-section [45,48]. One study even reports higher OPN levels in colostrum in milk from mothers who delivered via a C-section compared to vaginal delivery [52].

Breast milk from two different generations (1989 vs. 2013) in Japan showed a difference in the levels of OPN in colostrum; 318.1 mg/L (204.4–439.8) in 1989 vs. 137.5 mg/L (81.9–263.5) in 2013 [52]. All milk samples from 1989 and 2013 were stored at −80 °C within 12 h of collection, and the OPN concentrations were measured using the Quantikine Human Osteopontin ELISA (R&D Systems). Differences in exposure to sunlight, the method of childbirth and changes in maternal immune responses from the 1989 generation to the 2013 generation were suggested as possible explanations for this difference.

In conclusion, there seems to be a consensus on the level of OPN in human milk, at least among the largest studies using the Quantikine Human Osteopontin ELISA method validated for use in milk (R&D Systems). Most studies also show a decrease in OPN levels correlated with days postpartum. However, more studies are needed to establish maternal and other factors affecting the concentration of OPN in milk.

### 2.3. Quantification of OPN in Milk

The quantification of OPN in milk is made difficult by several factors. Firstly, OPN is heterogeneously modified by post-translational modification and proteolytic processing (described in later sections of this review). Secondly, OPN can form complexes with other components of the milk (e.g., lactoferrin, as described in a later section), which may also mask the epitopes so that they are not recognized by the antibodies, and hence, are not quantitated. Thirdly, the best ELISA method, the Quantikine Human Osteopontin ELISA (R&D Systems), is very sensitive, and milk samples must be diluted by a factor 10,000 to give absorption measurements in the dynamic range covered by the standard curve. This dilution procedure constitutes a significant source of error, which can translate into large differences in OPN concentrations among different studies. The MS/MS method used in some studies also has some important shortcomings. Most problematic is the selection of peptide(s) from OPN that are released quantitatively after tryptic digestion. As OPN is heavily and heterogeneously phosphorylated and glycosylated, and only contains a limited number of trypsin-cleavage sites, very few peptides (1–2 peptides) can be considered suitable to use. The peptide GDSVVYGLR is often used as a signature peptide in the quantification of OPN by MS/MS [45,48,50]. This peptide is present in both the intact protein and in the *N*-terminal fragments, which are abundantly present in milk (described in later sections of this review). Therefore, both full-length and *N*-terminal fragments are measured, but as the ratio between the full-length and *N*-terminal fragments is unknown in the individual milk samples, it is not possible correctly to convert the MS data to an OPN concentration. Furthermore, GDSVVYGLR can be phosphorylated by FAM20C [54,55], and therefore, it is not quantified.

In conclusion, no method alone can absolutely accurately determine the OPN concentration in milk. The most precise method consists of a combination of ELISA (or MS/MS quantification) with a method that can determine the degree of fragmentation of OPN in the milk (e.g., reverse-phase HPLC or gel filtration). However, since such analyses are very time- and labor-intensive, it is not possible to conduct them with large quantities of samples. We believe that the use of the Quantikine Human Osteopontin ELISA (R&D Systems), which is used by most groups today, provides the best estimate of OPN content in human milk samples.

## 3. Structure of Human and Bovine Milk Osteopontin

A single-copy gene on chromosome 4 encodes OPN, and during transcription, human OPN can undergo alternative splicing, generating two splice variants each lacking a single exon [56]. However, PCR analysis on a bovine mammary gland cDNA library with gene-specific primers showed a single transcript migrating, at a size corresponding to the expected size, without any alternative splicing [13]. Likewise, analyses of a human mammary gland cDNA library showed no alternative splicing, indicating that alternative splicing of OPN does not take place in bovine and human milk [13]. These findings are in line with other studies showing that alternative OPN splicing in human and bovine milk is not seen in normal specimens [57,58].

**Figure 1:**
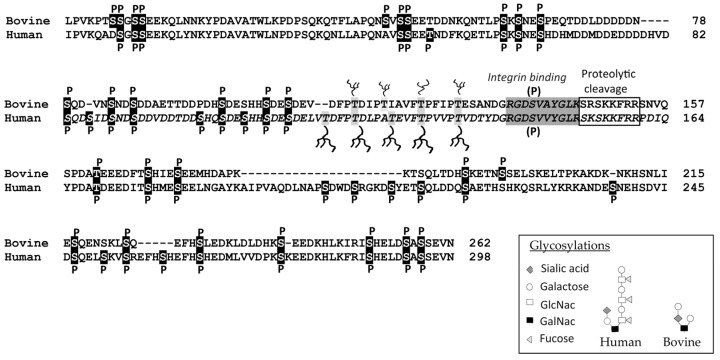
Alignment of bovine and human milk osteopontin (OPN). Phosphorylation and glycosylation sites identified in milk OPN are highlighted in black and grey, respectively [61,62]. The potential phosphorylation (P) of the SVAYGLK/SVVYGLR motif is also indicated [55]. The integrin-binding motifs are indicated in dark grey, and the regions containing the identified cleavage sites of OPN in milk are boxed [63,64]. The different glycan structures of OPN in bovine and human milk are indicated in the inserted box [13,65,66].

The mature human OPN protein comprises 298 amino acids, and its bovine counterpart contains 262 residues. The difference is mainly due to a missing sequence of 22 residues in bovine OPN, corresponding to residues 188–209 in human OPN (Figure 1). Human and bovine OPN sequences are highly homologous with identical amino acids on 182 positions, and an additional 44 residues are structurally conservative substitutions. OPN from both species has a very high content of aspartic and glutamic acid residues, and together with the considerable number of phosphorylated residues, this makes OPN a highly acidic protein. The pI value has been calculated to be 4.1, without taking phosphorylation into consideration. Isoelectric focusing of bovine milk OPN has shown migration corresponding to a pI of approximately 3.5 (Sørensen et al., 2007, unpublished data), which probably makes OPN the most acidic protein in milk. OPN contains two integrin-binding sequences, the Arg-Gly-Asp (RGD) sequence, and the adjacent cryptic SVVYGLR sequence (in bovine OPN: SVAYGLK), that bind specific integrins after it is exposed by proteolytic cleavage [59,60].

**Figure 2:**
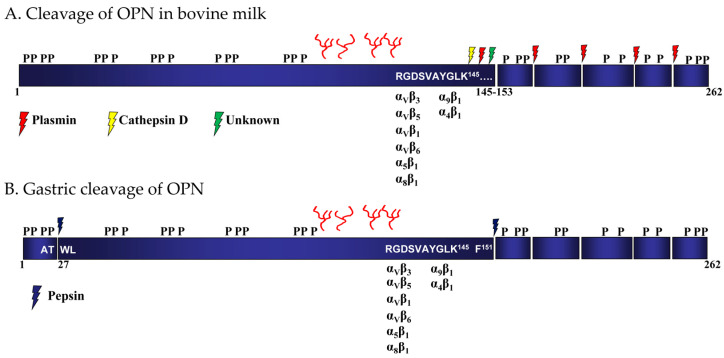
Proteolytic cleavage of bovine osteopontin (OPN) in milk and during gastric transit. The location of phosphorylation and *O*-glycosylation of bovine milk OPN is indicated [61]. The integrin-binding RGD and SVAYGLK motifs and the corresponding binding integrins are indicated. (**A**) Proteolytic cleavage of OPN in bovine milk [64] and (**B**) gastric cleavage of OPN by pepsin [74].

The phosphorylation patterns of bovine and human milk OPN have been thoroughly characterized. Bovine milk OPN contains, on average, 22 phosphorylated residues distributed over 28 different sites [61,65]. The degree of OPN phosphorylation in human milk has been shown to be even higher, with an average of ~25 phosphates distributed over 34 phosphoserines and two phosphothreonines [62,66]. A phosphoproteomics study has shown that OPN is the most phosphorylated protein in human colostrum and in the mature milk fat globule membrane [67]. The phosphorylations in OPN are predominately located in the recognition sequence of the kinase FAM20C (S-X-E/pS (phosphoserine)) [68]. FAM20C is localized in the Golgi lumen and is the main kinase phosphorylating secreted phosphoproteins [69]. This kinase, formerly known as mammary gland casein kinase or Golgi casein kinase [70,71], is responsible for the phosphorylation of all milk proteins, such as the caseins and PP3 [72,73]. The phosphorylated amino acids in both human and bovine milk OPN are grouped together in clusters of 3–5 phosphoresidues [61,62] (Figure 1 and Figure 2). The only part of OPN not containing phosphorylations is a threonine-proline rich region located just to the *N*-terminal side of the integrin-binding motifs. In this region, OPN contains several conserved threonine residues, of which 3–5 are modified by *O*-linked glycosylations in bovine and human milk OPN [61,62]. The glycan structures differ between human and bovine milk OPN, as the carbohydrates in human milk consists of fucosylated N-acetyllactosamine units [66], whereas a disialylated GalNAc-galactose core constitutes the glycans in bovine milk [13] (Figure 1). All mammalian OPN sequences contain one or more sequence motifs for N-linked glycosylation; however, N-linked glycosylation has not been shown in the milk OPN of any species.

In human and bovine milk, OPN is present as both a full-length protein and as different *N*-terminal-derived fragments resulting from endogenous proteolytic activity in the milk [10,57,63,64]. A conserved region of the OPN sequence next to the RGD and SVVYGLR (SVAYGLK in bovine OPN) motifs is particularly susceptible to proteolytic cleavage, as it contains many sites for proteases (plasmin, thrombin and cathepsin D) [63,64] (Figure 1 and Figure 2).

The *N*-terminal-derived fragments found in human milk are formed via cleavage of OPN by the endogenous proteases cathepsin D, and particularly plasmin, at Leu^151^-Arg^152^, Arg^152^-Ser^153^, Ser^153^-Lys^154^, Lys^154^-Ser^155^, Ser^155^-Lys^156^, Lys^156^-Lys^157^ or Phe^158^-Arg^159^ [63]. All *N*-terminal fragments generated by cleavage contain the integrin-binding ^143^RGD^145^-sequence, and most also contain the ^146^SVVYGLR^152^ motif. The corresponding *C*-terminal fragment has not been reported to be found in milk and is most likely further degraded to much smaller peptides by plasmin in both human and bovine milk [63,64] (Figure 2). In accordance with this, a total of 445 endogenous milk peptides were identified in human milk by LC-MS/MS, of which 61 peptides originated from OPN [75]. Only four peptides from the *N*-terminal were identified, whereas the remaining 57 peptides were derived from the *C*-terminal part of OPN. The identified peptides covered the entire *C*-terminal part of OPN, with plasmin predicted to cleave at the majority of the identified cleavage sites [75]. Likewise, no peptides originating from residues 29–153 in OPN were identified in a peptidomic analysis of human milk [76].

In conclusion, the sequences of human and bovine OPN are highly similar regarding their overall sequence identities, phosphorylation and glycosylation patterns, integrin-binding motifs and proteolytic cleavage sites.

## 4. Digestion and Uptake of Milk Osteopontin

Human and bovine milk OPN have been shown to remain intact after incubation with newborn stomach aspirates for one hour at a pH of 3 [77]. This is somewhat surprising, as OPN is an intrinsically disordered protein containing very little tertiary structure [78]; therefore, there are no steric hindrances for proteolytic enzymes to attack and digest the protein. However, milk OPN is extensively post-translationally modified, and these modifications play an important role in the digestion of the protein.

The location of the glycosylated and conserved threonine residues close to the important receptor-binding RGD motif suggests that the carbohydrates attached to OPN could function as protective structures against endogenous milk proteases and/or digestive enzymes. Indeed, the administration of exogenous bovine milk OPN, but not non-modified OPN, in the drinking water in a mouse colitis model reduced several disease parameters, indicating that OPN modification, such as glycosylation, has a protective effect against gastrointestinal digestion [79]. The protective role of the OPN glycosylation was confirmed in a study, showing that the glycosylated bovine OPN fragment Trp^27^-Phe^151^, containing the integrin-binding ^136^RGD^138^ and ^139^SVAYGLK^145^ motifs, resisted digestion with pepsin, whereas deglycosylated OPN was cleaved within the threonine/proline-rich region containing the glycosylations [74] (Figure 2). The generated fragment Trp^27^-Phe^151^ was capable of binding integrins via the RGD-sequence better than the full-length OPN protein. However, subsequent digestion with pancreatic proteases, simulating digestion in the small intestine, abolished its capability to bind integrins [74].

OPN fragments of a considerable size were identified using Western blots of OPN subjected to simulated gastrointestinal digestion with pepsin and pancreatin [80,81], indicating that a significant part of OPN resists digestion. However, it is also clear that large parts of OPN are digested, and peptides from both the *N*- and *C*-terminal parts of OPN have been identified by MS/MS analyses after in vitro simulated gastrointestinal digestion of human milk [82,83].

OPN has been shown to resist digestion in vivo, as biotinylated bovine milk OPN was detected via immunohistochemistry in the colons of mice fed bovine milk OPN through oral gavage [79]. Likewise, ELISA showed the presence of OPN in the plasma of knock-out mouse pups fed bovine milk OPN, indicating that OPN and/or fragments reached the circulation [79]. Similarly, OPN or OPN fragments were identified via competitive ELISA in the plasma of mice 1 to 4 h after they were fed bovine milk OPN [84]. After oral gavage, radioactively labeled OPN was detected in the livers and even the brains of mouse pups [29]. These studies illustrate that OPN or large OPN fragments can pass the intestinal barrier. The absorption of undigested or large OPN fragments has also been shown using Western blots of brain lysates from OPN knock-out mouse pups nursed by wildtype dams producing OPN-containing milk [29]. In a human intervention study, bovine milk OPN was identified by sandwich ELISA in the plasma of infants fed infant formula enriched with bovine milk OPN [47].

In an in vitro study, the bovine milk OPN fragment Trp^27^-Phe^151^, generated by pepsin cleavage, was shown to cross models of the intestinal barrier via transcytosis [85]. The naturally occurring *N*-terminal OPN fragments bound intestinal cells more effectively and were more effectively transported across the membrane models compared to full-length OPN. This is in accordance with earlier studies showing that the α_V_β_3_ integrin has a higher affinity for *N*-terminal fragments of OPN than the full-length OPN [63,64]. OPN co-localizes with the αvβ3 integrin on human intestinal crypt-like cells [81] and the Trp^27^-Phe^151^ fragment of OPN generated by pepsin also binds the α_V_β_3_ integrin in an RGD-dependent manner [74]. The intestinal binding of OPN or gastric digested OPN to the α_V_β_3_ integrin could potentially be involved in the transport of OPN across the intestinal barrier.

Collectively, these studies indicate that milk OPN is partly resistant to digestion by gut proteases. Since OPN is present in milk in relatively high concentrations, it can be expected that some of the ingested OPN reaches the intestine in a form that can bind integrins and potentially initiate signaling events. Studies also indicate that OPN- or RGD-containing OPN fragments are absorbed over the intestinal barrier and into the circulation, where they could potentially be involved in physiological processes.

## 5. Milk Osteopontin and Interaction with Integrins

Many of the functions mediated by OPN are initiated by interactions with cell surface receptors. The integrin family of receptors bind OPN’s core RGD motif, and they are the main receptor class for OPN binding [7,8,9]. OPN binds the α_V_β_1_, α_V_β_3_, α_V_β_5_, α_v_β_6_, α_5_β_1_ and α_8_β_1_ integrins via the RGD sequence [8,86], whereas the α_4_β_1_ and α_9_β_1_ integrins bind OPN through its cryptic SVVYGLR sequence [59,60]. In contrast, integrin α_X_β_2_ on myeloid leukocytes, and natural killer cells binds OPN through the negative charges on OPN independently of the RGD or SVVYGLR motifs [49,87].

The integrin-binding characteristics of OPN can change during proteolytic processing; e.g., thrombin cleavage at Arg^152^-Ser^153^ exposes the cryptic SVVYGLR motif of OPN, which is required for OPN binding to integrin α_9_β_1_ [60]. In milk, OPN is cleaved by plasmin at Lys^154^-Ser^155^, resulting in an *N*-terminal fragment that acts as a stronger ligand for the α5β_1_ and α_V_β_3_ integrins compared to full-length OPN [63]. In addition, the naturally occurring *N*-terminal fragments of OPN in human and bovine milk bind the α_V_β_3_ integrin more strongly than non-cleaved full-length OPNs [63,64]. The phosphorylation of milk OPN also affects its ability to bind integrins; since phosphorylated, but not dephosphorylated, OPN stimulates the production of interleukin-12 by macrophages via the β3-integrin [88]. Furthermore, phosphorylation of especially the *C*-terminal part of OPN has been shown to inhibit OPN binding to the α_V_β_3_ integrin [66]. After removal of the *C*-terminal part of OPN by plasmin in milk, or by pepsin in the stomach, during gastrointestinal transit, the capability of OPN to bind the α_V_β_3_ integrin is increased [66,74]. Atypical phosphorylation of the SVVYGLR motif in OPN by FAM20C, which occurs in both human and bovine mammary glands, also inhibits OPN interaction with the α_V_β_3_ integrin [55]. The FAM20C phosphorylation of the SVVYGLR motif could potentially also affect the engagement of OPN with the α_9_β_1_ integrin as small changes in the SVVYGLR motifs affect the interaction [89].

## 6. Effects of Milk Osteopontin on Intestinal Cells and Inflammatory Bowel Disease

OPN is a potent cytokine that plays a role in many inflammatory processes, and several studies have shown that the endogenous expression of OPN in immune cells or intestinal epithelial cells affects the status of the intestinal barrier in normal and diseased tissue. The endogenous expression of OPN in a normal gut indicates that it is involved in intestinal immune homeostasis [90], whereas lower expression in intestinal epithelial cells is linked to disruption of the epithelial barrier, as seen in Crohn’s disease. The level of OPN in plasma is related to the degree of inflammation in Crohn’s disease patients, and probably participates in the regulation of gastrointestinal immune reactions through its ability to stimulate T-cell cytokine production [91].

Regarding the effect of dietary OPN on inflammation of the digestive system, such as in colitis, the liver and the gut–liver axis have been investigated in several studies. An acute colitis mouse model (dextran sulfate sodium (DSS)-induced) showed that OPN knock-out mice had aggravated tissue destruction and weakened tissue repair compared to WT mice [92]. Later, it was shown that milk OPN administered in the drinking water was absorbed at the mucosal surface, and here, it mitigated the destructive effects of the induced colitis [79]. Specifically, the oral administration of milk OPN reduced the disease activity index and gut neutrophilic activity and increased the number of red blood cells compared to the DSS-treated mice that were not administered OPN. Furthermore, the expression of transforming growth factor beta-1 in the colon was higher, whereas the levels of pro-inflammatory cytokines were lower in OPN-treated mice [79]. This was a pioneering study, and to our knowledge, the first to indicate that orally administered milk OPN could alleviate intestinal inflammation. In a similar study investigating the effects of oral administration of bioactive milk components in a mouse model of DSS-induced colitis, OPN was shown to effectively lower the inflammatory score and myeloperoxidase activity that indicates neutrophil infiltration [93]. Further emphasizing the anti-inflammatory effect, a significant decrease in the numbers of T-cells, natural killer cells and dendritic cells and a significant decrease in cytokine expression were also seen in the mice administered the OPN-containing diet [93].

In an in vitro experiment, where T-cell cultures isolated from biopsies of Crohn’s disease patients were stimulated with bovine milk OPN, a dose-dependent bell-shaped increase in the production of IFN-γ, TNF-α and IL-10 was observed [91]. In T-cell cultures treated with OPN doses higher than 1 μg/mL, all three cytokines were downregulated [91]. This indicates that milk OPN can act both as a pro- and anti-inflammatory cytokine dependent on the dose. The concentration of OPN in milk is orders of magnitude higher than 1 μg/mL, and according to these data, such a concentration should result in downregulation of these inflammatory cytokines.

The oral administration of milk OPN has been shown to prevent the development of alcohol-induced liver injury in mice on an ethanol Lieber-DeCarli diet [94]. Mice fed an alcohol diet together with milk OPN showed increased gland height, crypt cell and enterocyte proliferation, and mucin content compared to mice fed an alcohol diet without milk OPN. In addition, the mice showed decreased levels of inflammation in the mucosa and submucosa of the gut membranes, evidenced by a lower presence of macrophages, lymphocytes and neutrophils. Furthermore, the milk OPN-treated mice preserved the expression of tight-junction proteins and showed reduced levels of translocation of Gram-negative bacteria, lipopolysaccharide levels and tumor necrosis factor-α than ethanol-fed mice. This indicates that dietary OPN helped counteract the detrimental effects of alcohol in the liver and gut [94].

In a study on preterm pigs prenatally exposed to LPS and subsequently fed diets with different bioactive milk components, one of them being OPN, no effects on crypt depth and villus height in the small intestines of preterm pigs were seen [95]. However, an increase in gut lactase activity was seen in pigs fed the OPN-containing diet. In a similar study, preterm pigs fed a raw bovine milk diet supplemented with bovine milk OPN were reported to have increased villus-to-crypt ratios in their small intestines compared to pigs fed a diet without added OPN [96]. Likewise, mouse pups nursed by OPN-knock-out dams had smaller villus height and crypt depth compared to pups receiving OPN from milk from wildtype dams or through a daily supplement of bovine milk OPN [97].

In summary, studies show that milk OPN affects intestinal cell cytokine production and gut cell morphology, including intestinal surface area, which can consequently enhance nutrient absorption; moreover, they show that milk OPN has mitigating effects on intestinal inflammatory diseases.

## 7. Milk Osteopontin and the Gut Transcriptome

Donovan et al. (2014) compared the intestinal transcriptomes of infant rhesus monkeys fed a standard milk-based formula, those fed a formula containing 125 mg/L bovine milk OPN and those nursed by their mothers [98]. Overall, differential expression of 1986 genes was seen among the three groups. In total, 1017 genes were differentially expressed between formula-fed monkeys and breast-nursed monkeys. When comparing the group receiving bovine milk OPN with the breast-fed monkeys, only 217 genes were differentially expressed. This demonstrated that the addition of bovine OPN to formula distinctly shifted the gene transcription to be more similar to that of breast-fed monkeys [98].

Recently, the effect of simulated gastrointestinal digested human and bovine milk OPN on gene expression in the intestinal-derived Caco-2 cell line was investigated [99]. Human and bovine milk OPN influenced the expression of 239 and 322 genes, respectively. For comparison, the milk protein α-lactalbumin had a very limited impact on gene expression, with only five differentially expressed genes identified. This emphasizes that the effect of OPN on intestinal cells is specific and that changes in the expressed genes are not merely an effect of added milk protein. Among the genes affected by the OPN treatments, 131 genes were regulated in a similar manner by human and bovine OPN. Analyses of the regulated genes showed that biological processes related to the ubiquitin system, DNA binding, and genes associated with transcription and transcription control pathways were affected by OPN [99].

Interestingly, the gene for ILF-2 (interleukin enhancer-binding factor 2) was shown to be upregulated in response to both human and bovine milk OPN [99]. ILF-2 is a transcription factor required for the T-cell expression of interleukin-2 [100], which is involved in oral tolerance and immunity through interactions with T-cells [101]. These findings are in accordance with a large intervention study showing that human infants fed formula supplemented with bovine milk OPN had similar plasma levels of interleukin-2 to breast-fed infants [102]. Collectively, the upregulation of ILF-2 in both studies demonstrates that OPN can affect the expression of interleukin-2 from T-cells.

In a study comparing the transcriptional effects of lactoferrin from human and bovine milk on human intestinal epithelial crypt-like cells, it was shown that 29 differentially expressed genes were regulated by both human and bovine lactoferrin (out of 150 and 350 genes regulated by human and bovine lactoferrin, respectively) [103]. This suggests that the substitution of human OPN with bovine OPN is more conservative regarding the influence on gene expression (with 55% of the genes regulated by human OPN also being regulated by bovine OPN) than the replacement of human lactoferrin with bovine lactoferrin.

In conclusion, these transcriptome studies show that milk OPN has a significant effect on the transcription of genes in intestinal cells, though these studies must be interpreted with caution, as it is not clear whether the identified mRNAs will result in translated proteins.

## 8. Milk Osteopontin and the Gut Microbiome

The effect of endogenously expressed OPN on the gut microbiota has been addressed in several studies. The role of OPN in the development of colitis showed that the enteric bacterial profile of OPN/IL-10 double knock-out mice is distinctly different from that of IL-10 KO mice, indicating that OPN expression in enteric epithelial cells affects the microbiota [104]. Specifically, OPN knock-out mice showed a significantly lower abundance of *Clostridium* subcluster XIVa and a greater abundance of *Clostridium* cluster XVIII [104]. The role of *Clostridium* cluster XVIII in gut mucosal immunity is not clear, whereas *Clostridium* subcluster XIVa has a protective effect on experimentally induced colitis in murine models via the induction of regulatory T lymphocytes in the large bowel [105]. In a study on acute graft-versus-host disease, T-cell-derived OPN played a protective role by modulating the gut microbiome by increasing the levels of the commensal bacteria *Akkermansia* [106]. Furthermore, secreted OPN lowered the levels of commensal bacteria of the *Bacteroidales* order.

Several studies have investigated the effect of orally administered milk OPN on the microbiota. Milk OPN has been shown to bind bacterial lipopolysaccharide in mice treated with an alcoholic diet, thereby lowering tumor necrosis factor-α expression and the subsequent development of alcohol-induced liver damage [94,107]. An investigation of the underlying mechanism showed that milk OPN counteracts the alcohol-induced reduction in the expression of the antimicrobial peptides Reg3b and Reg3g, which regulates bacterial growth in the intestine [108]. It was shown that OPN stimulates the growth of tryptophan-metabolizing and short-chain fatty acid (SCFA)-synthesizing bacteria, including *Bifidobacterium*, *Eubacterium*, *Prevotella*, *Allloprevotella*, *Desulfovibrio*, *Butyricicoccus*, *Butyricimonas* and *Roseburia*. These bacteria are considered beneficial for gut health, as they produce SCFAs from undigested carbohydrates, which increases expression of tight junction proteins in the intestinal membrane. This preserves gut barrier function and limits the access of bacteria and bacterial products, such as LPS, from the gut to the portal blood.

In mice fed a high-fat diet, OPN intensified lipid accumulation and metabolic disorders [109]. Interestingly, OPN induced changes in the gut microbiome, and feeding with milk OPN led to a higher abundance of *Dorea* but fewer *Lactobacillus* (the study used the genus of *Lactobacillus* from before the taxonomic revision of the *Lactobacillus* genus in 2020), which were found to be positively and negatively correlated with body weight, respectively. OPN was suggested to control the amount of *Lactobacillus* by decreasing the adhesion of *Lactobacillus* to epithelial cells in the intestine through the Notch signaling pathway [109].

Enterotoxigenic *Escherichia coli* (F-18 strain)-challenged pigs fed recombinant algae expressing OPN showed a different microbiome compared to pigs receiving algae not expressing OPN [110]. The ingestion of OPN-enriched algal protein elicited shifts in bacterial α-diversity, and the pigs receiving the algae OPN diet showed a reduction in *Firmicutes* and *Bacteroidetes* and an increase in the *Streptococcus* and *Blautia* genera. In the phylum *Bacteroidetes*, decreased abundance was observed for the following genera: *Rikenellaceae*, the RC9 gut group, the dgA-11 gut group, an uncultured bacterium genus of the *Muribaculaceae* family, and three genera of the *Prevotellaceae* family (*Prevotella* 2, *Prevotella* 7 and *Prevotella* 1). Genera within the *Firmicutes* phylum (*Candidatus*, *Soleaferrea* and *Lachnospiraceae*) were also decreased in pigs consuming the OPN-enriched algal protein [110].

In summary, dietary OPN has the potential to affect the gut microbiome, but as studies point in different directions regarding which bacteria are influenced and how this translates into physiological effects, more controlled studies are needed to draw conclusions on the effect of OPN on the microbiota.

## 9. Milk Osteopontin and Immunological Effects

In 1989, Patarca et al. described a murine cDNA, named Early T lymphocyte activation 1 (ETA-1), which was highly expressed after the activation of T-cells [4]. The cDNA was a murine homologue of rat bone OPN identified three years earlier [3]. It has been known for a long time that OPN is involved in immune processes and expressed by many different types of immune cells [111]. The concentration of OPN in milk is orders of magnitude higher than anywhere else in the body, which could indicate that OPN plays a role either as part of the innate immune system in milk, or as a modulator of immune responses in neonates and infants.

Bovine milk OPN has been shown to induce the expression of the T helper-1 cytokine IL-12 in cultured human lamina propria mononuclear cells isolated from intestinal biopsies [36], indicating that milk OPN could induce cytokine production in neonate intestinal immune cells. Milk OPN binds monocytes, but not resting T-cells, NK cells or B-cells, and mediates the chemoattraction of IL-1-activated human monocytes [49]. OPN was shown to bind all known serotypes of the two bacterial species *Streptococcus agalactiae* and *Staphylococcus aureus* and to opsonize these bacteria for phagocytosis [49]. In another in vitro assay, sterile skimmed milk from Jersey cows supplemented with bovine OPN showed antibacterial activity in a dose-dependent manner by inhibiting the growth of *S. epidermidis* [96], a common neonatal pathogen causing late-onset sepsis following gut translocation [112]. Collectively, these studies show that milk OPN could play an important role as an inducer of cytokine production in the infant intestine, as an opsonin marking bacteria for removal by macrophages or as an antibacterial agent targeting specific pathogenic bacteria.

Several studies have investigated the immunological activities of milk OPN in vivo. In a clinical trial performed in Shanghai, China, children of 1 to 6 months of age were randomized into groups receiving either regular formula or the same formula with bovine OPN at 65 mg/mL or 130 mg/L (corresponding to 50% and 100% of human milk levels, respectively), and were compared with a reference group of breast-fed infants [102]. The pro-inflammatory cytokine TNF-α was found to be higher in plasma from formula-fed infants than in infants receiving breast milk. However, among the formula-fed groups, the levels of TNF-α were significantly lower in groups receiving formulas fortified with OPN, suggesting that bovine milk OPN downregulates inflammatory cytokines in human infants. The study also showed higher levels of interleukin-2 in the OPN-fortified groups compared to the standard formula group at four months of age, and that OPN shifted the infants’ amino-acid metabolism and cytokine responses towards those of breast-fed infants. Most interestingly, infants receiving the OPN-fortified formulas had fewer days with fever (pyrexia) than those receiving formula without added OPN, strongly indicating that milk OPN had an effect on the immune systems of the infants [102]. Analyses of plasma from the infants showed that the proportion of circulating T-cells in the group receiving 130 mg/L OPN was higher compared with the groups receiving regular formula or formula with only 65 mg/L OPN added, further substantiating that OPN affects immune developmental processes in infants [113]. Interestingly, in relation to OPN and pyrexia, a correlation with days of hospitalization due to fever-related illness in the first three months post-partum was also reported for infants of mothers who had high concentrations of OPN in their breast milk compared to infants of mothers with lower levels of breast milk OPN [46].

The effect of ingested milk OPN on immunity has also been investigated in a preterm pig model delivered via cesarean section at 90% gestation. The pigs were fed milk OPN at a dose of 46 mg/kg body weight per day, and their clinical outcomes were compared to those of pigs fed raw bovine milk. The study showed that most immune parameters were similar between the OPN and the control groups. However, the OPN pigs had higher intestinal villus-to-crypt ratios than pigs fed the milk control, and higher monocyte and lymphocyte counts on day 8 [96].

In summary, there is good evidence that the intake of milk OPN has an influence on immune processes in the gut, and can thereby affect the health of, for example, infants who consume milk OPN.

## 10. Milk Osteopontin’s Effects on Brain Development and Cognitive Function

OPN is expressed in the brainstem, cerebellum and amoeboid microganglia in developing rat brains [114,115]. OPN is also shown to mediate myelination in mice [116], and most interestingly, the injection of exogenous OPN induced endogenous OPN expression in rats exposed to hypoxic–ischemic injury and improved neurologic outcomes [117]. These and other studies suggest that OPN plays a role in brain development. As OPN is present in high concentrations in milk, it was hypothesized that milk OPN could influence brain development, and hence, cognitive behavior, potentially contributing to improved cognitive function in breast-fed versus formula-fed infants [118].

In a knock-out model, it was shown that OPN wildtype mouse pups nursed by OPN knock-out dams had significantly less OPN protein in their brains compared to mice nursed by wildtype dams. Most interestingly, this difference translated into impaired learning and memory abilities in these mouse pups [29,97]. Furthermore, a higher level of expression of myelination-related proteins and increased differentiation and proliferation of glia cells into oligodendrocytes in the brains of pups nursed by wildtype mice were observed [29]. This suggests that the improved cognitive capabilities of the mice can be ascribed to OPN improving the myelination processes in the brains of the mouse pups. This is in accordance with a recent in vitro study showing that OPN stimulated the maturation, proliferation and differentiation of oligodendrocyte progenitor cells into mature oligodendrocytes [119].

In a pig model, the effect on recognition memory was tested in two groups of pigs (postnatal days 2–34) provided with an ad libitum diet of either a standard soy protein isolate diet or the same diet supplemented with bovine milk OPN [120]. Pigs fed the OPN-containing diet showed shorter latency to the first object visited compared to pigs in the control group, but no difference in the recognition index between dietary groups was observed. Interestingly, the neuroimaging outcomes showed an increased volume of several brain regions in the pigs receiving the OPN diet. However, in another pig study using preterm pigs delivered via cesarean section (90% gestation), the addition of OPN to their diet (46 mg/kg per day) showed no effects on cognitive performance [96].

Collectively, these studies suggest that dietary milk OPN could play a role in cellular processes in the brain, potentially affecting cognitive development and behavior, but more research is needed to elucidate this.

## 11. Osteopontin–Lactoferrin Complex

OPN has been shown to form complexes with lactoferrin and lactoperoxidase in bovine milk through electrostatic interactions, and with IgM through affinity interactions [31]. The interaction between bovine milk OPN and lactoferrin was shown to have an dissociation constant of 10^−6^ M, and the complexes were formed in a 3:1 ratio (Lactoferrin:OPN) [121]. Human OPN and lactoferrin also form complexes in vitro [80]. The lactoferrin–OPN complex has been shown to be more resistant to gastrointestinal digestion than the individual proteins alone [80,81], and the complex formation increases binding and uptake by human intestinal cells [80]. The complex co-localizes with the main OPN receptor, the α_V_β_3_ integrin, on intestinal cells, and the proteins are internalized as a complex [81]. Both bovine and human complexes promote the proliferation and differentiation of human intestinal cells and enhance intestinal immunity by stimulating the expression of IL-18 [80,81,122]. The bioactivities of the bovine lactoferrin–OPN complex are also seen when it is added in formula protein blends [122]. Interestingly, the ratio between the concentration of the two proteins is the same in bovine (~20 mg/L OPN, ~200 mg/L lactoferrin) and human milk (~200 mg/L OPN, ~2000 mg/L lactoferrin) [18].

## 12. Milk Osteopontin and Dental Health

Endogenously expressed OPN has been localized to non-mineralized tissues during tooth development, and has been suggested to function as an inhibitor of mineralization and/or as a mediator of cell–matrix and matrix–matrix/mineral adhesion during the formation and turnover of mineralized tissues [123,124].

The structural properties of OPN, containing many acidic and phosphorylated residues capable of binding anions, has led to the idea that milk OPN could be used to deliver calcium ions to the mineralizing tooth surface. Recently, OPN was shown to increase the remineralization of demineralized enamel lesions in the presence of calcium phosphate and fluoride [125].

OPN binds to a variety of different bacteria, and it has been shown to act as an opsonin that promotes macrophage phagocytosis [49]. In the mouth, it has been proposed that the affinity of OPN for bacteria be exploited in new therapeutic products for oral hygiene [126]. The administration of bovine milk OPN has been shown to decrease the bacterial adhesion of *Actinomyces viscosus, Lacticaseibacillus paracasei* subsp. *Paracasei*, *Staphylococcus epidermidis*, *Streptococcus oralis*, *Streptococcus mitis*, *Streptococcus sanguinis*, *Streptococcus downei* and *Actinomyces naeslundii* and to delay biofilm formation on teeth, and may reduce the occurrence of dental caries or periodontal disease [127,128,129]. Recently, Kristensen et al. showed that milk OPN was effective in reducing the adhesion *of Actinomyces naeslundii*, *Lacticaseibacillus paracasei* subsp. *paracasei* and *Streptococcus mitis* to saliva-coated surfaces, mimicking the tooth enamel [130].

## 13. Conclusions

In conclusion, the levels of OPN in human milk are reported to be 48–334 mg/L, depending on time postpartum and the geographical location of the mothers. Most studies show a decrease in OPN levels that is correlated with days postpartum. However, more studies are needed to establish maternal and other factors affecting the concentration of OPN in milk.

The structures of human and bovine milk OPN are highly similar regarding their overall sequence identities, phosphorylation and glycosylation patterns, integrin-binding motifs, and proteolytic cleavage sites. Milk OPN is partly resistant to digestion by gut proteases, and it is likely that some of the ingested OPN reaches the intestine in a form that can bind integrins and potentially initiate signaling events. Several studies have also shown that OPN or RGD-containing OPN fragments are absorbed over the intestinal barrier and into the circulation and organs, where they can potentially be involved in physiological processes.

Milk OPN induces intestinal cell cytokine production and affects the development and cellular morphology of the intestines. Furthermore, several studies have indicated milk OPN’s mitigating effects on intestinal inflammatory diseases. Dietary milk OPN affects the transcription of genes in the intestine and influences the gut microbiome. Finally, milk OPN improves immune parameters in infants fed OPN-containing formula and has been shown to affect cognitive development and behavior in animal models.

## Data Availability

Not applicable.

## References

[1] Senger D.R., Wirth D.F., Hynes R.O. (1979). Transformed Mammalian Cells Secrete Specific Proteins and Phosphoproteins. Cell.

[2] Franzén A., Heinegård D. (1985). Isolation and Characterization of Two Sialoproteins Present Only in Bone Calcified Matrix. Biochem. J..

[3] Oldberg A., Franzén A., Heinegård D. (1986). Cloning and Sequence Analysis of Rat Bone Sialoprotein (Osteopontin) CDNA Reveals an Arg-Gly-Asp Cell-Binding Sequence. Proc. Natl. Acad. Sci. USA.

[4] Patarca R., Freeman G.J., Singh R.P., Wei F.Y., Durfee T., Blattner F., Regnier D.C., Kozak C.A., Mock B.A., Morse H.C. (1989). Structural and Functional Studies of the Early T Lymphocyte Activation 1 (Eta-1) Gene. Definition of a Novel T Cell-Dependent Response Associated with Genetic Resistance to Bacterial Infection. J. Exp. Med..

[5] Senger D.R., Perruzzi C.A., Papadopoulos A. (1989). Elevated Expression of Secreted Phosphoprotein I (Osteopontin, 2ar) as a Consequence of Neoplastic Transformation. Anticancer Res..

[6] Shiraga H., Min W., VanDusen W.J., Clayman M.D., Miner D., Terrell C.H., Sherbotie J.R., Foreman J.W., Przysiecki C., Neilson E.G. (1992). Inhibition of Calcium Oxalate Crystal Growth in Vitro by Uropontin: Another Member of the Aspartic Acid-Rich Protein Superfamily. Proc. Natl. Acad. Sci. USA.

[7] Sodek J., Ganss B., McKee M.D. (2000). Osteopontin. Crit. Rev. Oral Biol. Med..

[8] Icer M.A., Gezmen-Karadag M. (2018). The Multiple Functions and Mechanisms of Osteopontin. Clin. Biochem..

[9] Lok Z.S.Y., Lyle A.N. (2019). Osteopontin in Vascular Disease. Arterioscler. Thromb. Vasc. Biol..

[10] Senger D.R., Perruzzi C.A., Papadopoulos A., Tenen D.G. (1989). Purification of a Human Milk Protein Closely Similar to Tumor-Secreted Phosphoproteins and Osteopontin. Biochim. Biophys. Acta BBA-Protein Struct. Mol. Enzymol..

[11] Sørensen E.S., Petersen T.E. (1993). Purification and Characterization of Three Proteins Isolated from the Proteose Peptone Fraction of Bovine Milk. J. Dairy Res..

[12] Turck D., Castenmiller J., De Henauw S., Hirsch-Ernst K.I., Kearney J., Maciuk A., Mangelsdorf I., McArdle H.J., Naska A., EFSA Panel on Nutrition, Novel Foods and Food Allergens (NDA) (2022). Safety of Bovine Milk Osteopontin as a Novel Food Pursuant to Regulation (EU) 2015/2283. EFSA J..

[13] Christensen B., Sørensen E.S. (2016). Structure, Function and Nutritional Potential of Milk Osteopontin. Int. Dairy J..

[14] Demmelmair H., Prell C., Timby N., Lönnerdal B. (2017). Benefits of Lactoferrin, Osteopontin and Milk Fat Globule Membranes for Infants. Nutrients.

[15] Jia Q., Wang Y., Zhu J., Yu H., Tong X. (2021). A Literature Review on Lactopontin and Its Roles in Early Life. Transl. Pediatr..

[16] Jiang R., Lönnerdal B. (2016). Biological Roles of Milk Osteopontin. Curr. Opin. Clin. Nutr. Metab. Care.

[17] Jiang R., Lönnerdal B. (2020). Effects of Milk Osteopontin on Intestine, Neurodevelopment, and Immunity. Nestle Nutr. Inst. Workshop Ser..

[18] Levy E., Marcil V., Tagharist Ép Baumel S., Dahan N., Delvin E., Spahis S. (2023). Lactoferrin, Osteopontin and Lactoferrin-Osteopontin Complex: A Critical Look on Their Role in Perinatal Period and Cardiometabolic Disorders. Nutrients.

[19] Auestad N., Layman D.K. (2021). Dairy Bioactive Proteins and Peptides: A Narrative Review. Nutr. Rev..

[20] Bruni N., Capucchio M., Biasibetti E., Pessione E., Cirrincione S., Giraudo L., Corona A., Dosio F. (2016). Antimicrobial Activity of Lactoferrin-Related Peptides and Applications in Human and Veterinary Medicine. Molecules.

[21] Lenton S., Nylander T., Holt C., Sawyer L., Härtlein M., Müller H., Teixeira S.C.M. (2016). Structural Studies of Hydrated Samples of Amorphous Calcium Phosphate and Phosphoprotein Nanoclusters. Eur. Biophys. J..

[22] Bielecka M., Cichosz G., Czeczot H. (2022). Antioxidant, Antimicrobial and Anticarcinogenic Activities of Bovine Milk Proteins and Their Hydrolysates—A Review. Int. Dairy J..

[23] Hernández-Ledesma B., Recio I., Amigo L. (2008). β-Lactoglobulin as Source of Bioactive Peptides. Amino Acids.

[24] Kamau S.M., Cheison S.C., Chen W., Liu X.-M., Lu R.-R. (2010). Alpha-Lactalbumin: Its Production Technologies and Bioactive Peptides. Compr. Rev. Food Sci. Food Saf..

[25] Nielsen S.D., Beverly R.L., Qu Y., Dallas D.C. (2017). Milk Bioactive Peptide Database: A Comprehensive Database of Milk Protein-Derived Bioactive Peptides and Novel Visualization. Food Chem..

[26] Cleland T.P., Power M.L. (2022). Variation in Milk Proteins Across Lactation in *Pongo pygmaeus* and *Gorilla gorilla*. J. Proteome Res..

[27] Hu B., Zhang J., Jiang Y., Tong W., Lai S., Ren Y. (2021). Quantitative Determination of Osteopontin in Bovine, Buffalo, Yak, Sheep and Goat Milk by Ultra-High Performance Liquid Chromatography-Tandem Mass Spectrometry and Stable Isotope Dimethyl Labeling. Food Chem..

[28] Zou Z., Duley J.A., Cowley D.M., Reed S., Arachchige B.J., Bhandari B., Shaw P.N., Bansal N. (2022). Physicochemical Properties and Whey Proteomes of Camel Milk Powders Produced by Different Concentration and Dehydration Processes. Foods.

[29] Jiang R., Prell C., Lönnerdal B. (2019). Milk Osteopontin Promotes Brain Development by Up-Regulating Osteopontin in the Brain in Early Life. FASEB J..

[30] Bayless K.J., Davis G.E., Meininger G.A. (1997). Isolation and Biological Properties of Osteopontin from Bovine Milk. Protein Expr. Purif..

[31] Azuma N., Maeta A., Fukuchi K., Kanno C. (2006). A Rapid Method for Purifying Osteopontin from Bovine Milk and Interaction between Osteopontin and Other Milk Proteins. Int. Dairy J..

[32] Bertelsen H., Wejse P.L., Trúgvason T. (2015). Method for Isolating Osteopontin Using Concentrated Feeds. U.S. Patent.

[33] Kvistgaard A.S., Matulka R.A., Dolan L.C., Ramanujam K.S. (2014). Pre-Clinical in Vitro and in Vivo Safety Evaluation of Bovine Whey Derived Osteopontin, Lacprodan^®^ OPN-10. Food Chem. Toxicol..

[34] Sørensen E.S., Ostersen S., Chatterton D.E.W., Holst H.H., Albertsen K. (2007). Process for Isolation of Osteopontin from Milk. U.S. Patent.

[35] Christensen B., Zachariae E.D., Poulsen N.A., Buitenhuis A.J., Larsen L.B., Sørensen E.S. (2021). Factors Influencing Milk Osteopontin Concentration Based on Measurements from Danish Holstein Cows. J. Dairy Res..

[36] Schack L., Lange A., Kelsen J., Agnholt J., Christensen B., Petersen T.E., Sørensen E.S. (2009). Considerable Variation in the Concentration of Osteopontin in Human Milk, Bovine Milk, and Infant Formulas. J. Dairy Sci..

[37] Dudemaine P.L., Thibault C., Alain K., Bissonnette N. (2014). Genetic Variations in the SPP1 Promoter Affect Gene Expression and the Level of Osteopontin Secretion into Bovine Milk. Anim. Genet..

[38] Bissonnette N. (2018). Short Communication: Genetic Association of Variations in the Osteopontin Gene (SPP1) with Lactation Persistency in Dairy Cattle. J. Dairy Sci..

[39] Khatib H., Zaitoun I., Wiebelhaus-Finger J., Chang Y.M., Rosa G.J.M. (2007). The Association of Bovine PPARGC1A and OPN Genes with Milk Composition in Two Independent Holstein Cattle Populations. J. Dairy Sci..

[40] Leonard S., Khatib H., Schutzkus V., Chang Y.M., Maltecca C. (2005). Effects of the Osteopontin Gene Variants on Milk Production Traits in Dairy Cattle. J. Dairy Sci..

[41] Nagatomo T., Ohga S., Takada H., Nomura A., Hikino S., Imura M., Ohshima K., Hara T. (2004). Microarray Analysis of Human Milk Cells: Persistent High Expression of Osteopontin during the Lactation Period. Clin. Exp. Immunol..

[42] Bruun S., Jacobsen L.N., Ze X., Husby S., Ueno H.M., Nojiri K., Kobayashi S., Kwon J., Liu X., Yan S. (2018). Osteopontin Levels in Human Milk Vary Across Countries and Within Lactation Period: Data From a Multicenter Study. J. Pediatr. Gastroenterol. Nutr..

[43] Ruan H., Tang Q., Zhao X., Zhang Y., Zhao X., Xiang Y., Geng W., Feng Y., Cai W. (2022). The Levels of Osteopontin in Human Milk of Chinese Mothers and Its Associations with Maternal Body Composition. Food Sci. Hum. Wellness.

[44] Zhou Y., Chen Q., Jiang R., Wang J., Duan Y., Bi Y., Yang Z., Lai J. (2022). Concentration of osteopontin in human milk and associated factors in Chinese populations from 2011 to 2013. Wei Sheng Yan Jiu.

[45] Zhu J., Yu X., Wang Y., Bai S., Lai J., Tong X., Xing Y. (2022). Longitudinal Changes of Lactopontin (Milk Osteopontin) in Term and Preterm Human Milk. Front. Nutr..

[46] Aksan A., Erdal I., Yalcin S.S., Stein J., Samur G. (2021). Osteopontin Levels in Human Milk Are Related to Maternal Nutrition and Infant Health and Growth. Nutrients.

[47] Jiang R., Lönnerdal B. (2019). Osteopontin in Human Milk and Infant Formula Affects Infant Plasma Osteopontin Concentrations. Pediatr. Res..

[48] Zhang J., Zhao A., Lai S., Yuan Q., Jia X., Wang P., Zhang Y. (2021). Longitudinal Changes in the Concentration of Major Human Milk Proteins in the First Six Months of Lactation and Their Effects on Infant Growth. Nutrients.

[49] Schack L., Stapulionis R., Christensen B., Kofod-Olsen E., Skov Sørensen U.B., Vorup-Jensen T., Sørensen E.S., Höllsberg P. (2009). Osteopontin Enhances Phagocytosis through a Novel Osteopontin Receptor, the AlphaXbeta2 Integrin. J. Immunol..

[50] Goonatilleke E., Huang J., Xu G., Wu L., Smilowitz J.T., German J.B., Lebrilla C.B. (2019). Human Milk Proteins and Their Glycosylation Exhibit Quantitative Dynamic Variations during Lactation. J. Nutr..

[51] Liang N., Koh J., Kim B.J., Ozturk G., Barile D., Dallas D.C. (2022). Structural and Functional Changes of Bioactive Proteins in Donor Human Milk Treated by Vat-Pasteurization, Retort Sterilization, Ultra-High-Temperature Sterilization, Freeze-Thawing and Homogenization. Front. Nutr..

[52] Takahashi T., Ueno H.M., Yamaide F., Nakano T., Shiko Y., Kawasaki Y., Mitsuishi C., Shimojo N. (2023). Comparison of 30 Cytokines in Human Breast Milk between 1989 and 2013 in Japan. Nutrients.

[53] Ge B., Liu H., Liang Q., Shang L., Wang T., Ge S. (2019). Oxytocin Facilitates the Proliferation, Migration and Osteogenic Differentiation of Human Periodontal Stem Cells in Vitro. Arch. Oral Biol..

[54] Mateos B., Holzinger J., Conrad-Billroth C., Platzer G., Żerko S., Sealey-Cardona M., Anrather D., Koźmiński W., Konrat R. (2021). Hyperphosphorylation of Human Osteopontin and Its Impact on Structural Dynamics and Molecular Recognition. Biochemistry.

[55] Schytte G.N., Christensen B., Bregenov I., Kjøge K., Scavenius C., Petersen S.V., Enghild J.J., Sørensen E.S. (2020). FAM20C Phosphorylation of the RGDSVVYGLR Motif in Osteopontin Inhibits Interaction with the Avβ3 Integrin. J. Cell. Biochem..

[56] Young M.F., Kerr J.M., Termine J.D., Wewer U.M., Wang M.G., McBride O.W., Fisher L.W. (1990). CDNA Cloning, MRNA Distribution and Heterogeneity, Chromosomal Location, and RFLP Analysis of Human Osteopontin (OPN). Genomics.

[57] Bissonnette N., Dudemaine P.L., Thibault C., Robitaille G. (2012). Proteomic Analysis and Immunodetection of the Bovine Milk Osteopontin Isoforms. J. Dairy Sci..

[58] Mirza M., Shaughnessy E., Hurley J.K., Vanpatten K.A., Pestano G.A., He B., Weber G.F. (2008). Osteopontin-c Is a Selective Marker of Breast Cancer. Int. J. Cancer.

[59] Bayless K.J., Davis G.E. (2001). Identification of Dual Alpha 4beta1 Integrin Binding Sites within a 38 Amino Acid Domain in the *N*-Terminal Thrombin Fragment of Human Osteopontin. J. Biol. Chem..

[60] Yokosaki Y., Matsuura N., Sasaki T., Murakami I., Schneider H., Higashiyama S., Saitoh Y., Yamakido M., Taooka Y., Sheppard D. (1999). The Integrin Alpha(9)Beta(1) Binds to a Novel Recognition Sequence (SVVYGLR) in the Thrombin-Cleaved Amino-Terminal Fragment of Osteopontin. J. Biol. Chem..

[61] Sørensen E.S., Højrup P., Petersen T.E. (1995). Posttranslational Modifications of Bovine Osteopontin: Identification of Twenty-Eight Phosphorylation and Three O-Glycosylation Sites. Protein Sci..

[62] Christensen B., Nielsen M.S., Haselmann K.F., Petersen T.E., Sørensen E.S. (2005). Post-Translationally Modified Residues of Native Human Osteopontin Are Located in Clusters: Identification of 36 Phosphorylation and Five O-Glycosylation Sites and Their Biological Implications. Biochem. J..

[63] Christensen B., Schack L., Kläning E., Sørensen E.S. (2010). Osteopontin Is Cleaved at Multiple Sites Close to Its Integrin-Binding Motifs in Milk and Is a Novel Substrate for Plasmin and Cathepsin D. J. Biol. Chem..

[64] Christensen B., Sørensen E.S. (2014). Osteopontin Is Highly Susceptible to Cleavage in Bovine Milk and the Proteolytic Fragments Bind the AVβ₃-Integrin Receptor. J. Dairy Sci..

[65] Boskey A.L., Christensen B., Taleb H., Sørensen E.S. (2012). Post-Translational Modification of Osteopontin: Effects on in Vitro Hydroxyapatite Formation and Growth. Biochem. Biophys. Res. Commun..

[66] Christensen B., Kläning E., Nielsen M.S., Andersen M.H., Sørensen E.S. (2012). *C*-Terminal Modification of Osteopontin Inhibits Interaction with the AVβ3-Integrin. J. Biol. Chem..

[67] Yang M., Deng W., Cao X., Wang L., Yu N., Zheng Y., Wu J., Wu R., Yue X. (2020). Quantitative Phosphoproteomics of Milk Fat Globule Membrane in Human Colostrum and Mature Milk: New Insights into Changes in Protein Phosphorylation during Lactation. J. Agric. Food Chem..

[68] Tagliabracci V.S., Engel J.L., Wen J., Wiley S.E., Worby C.A., Kinch L.N., Xiao J., Grishin N.V., Dixon J.E. (2012). Secreted Kinase Phosphorylates Extracellular Proteins That Regulate Biomineralization. Science.

[69] Tagliabracci V.S., Wiley S.E., Guo X., Kinch L.N., Durrant E., Wen J., Xiao J., Cui J., Nguyen K.B., Engel J.L. (2015). A Single Kinase Generates the Majority of the Secreted Phosphoproteome. Cell.

[70] Lasa-Benito M., Marin O., Meggio F., Pinna L.A. (1996). Golgi Apparatus Mammary Gland Casein Kinase: Monitoring by a Specific Peptide Substrate and Definition of Specificity Determinants. FEBS Lett..

[71] Mercier J.-C. (1981). Phosphorylation of Caseins, Present Evidence for an Amino Acid Triplet Code Posttranslationally Recognized by Specific Kinases. Biochimie.

[72] Fang Z.H., Visker M.H.P.W., Miranda G., Delacroix-Buchet A., Bovenhuis H., Martin P. (2016). The Relationships among Bovine AS-Casein Phosphorylation Isoforms Suggest Different Phosphorylation Pathways. J. Dairy Sci..

[73] Sørensen E.S., Petersen T.E. (1993). Phosphorylation, Glycosylation and Amino Acid Sequence of Component PP3 from the Proteose Peptone Fraction of Bovine Milk. J. Dairy Res..

[74] Christensen B., Karlsen N.J., Jørgensen S.D.S., Jacobsen L.N., Ostenfeld M.S., Petersen S.V., Müllertz A., Sørensen E.S. (2020). Milk Osteopontin Retains Integrin-Binding Activity after in Vitro Gastrointestinal Transit. J. Dairy Sci..

[75] Dallas D.C., Smink C.J., Robinson R.C., Tian T., Guerrero A., Parker E.A., Smilowitz J.T., Hettinga K.A., Underwood M.A., Lebrilla C.B. (2015). Endogenous Human Milk Peptide Release Is Greater after Preterm Birth than Term Birth. J. Nutr..

[76] Wada Y., Lönnerdal B. (2014). Bioactive Peptides Derived from Human Milk Proteins—Mechanisms of Action. J. Nutr. Biochem..

[77] Chatterton D.E.W., Rasmussen J.T., Heegaard C.W., Sørensen E.S., Petersen T.E. (2004). In Vitro Digestion of Novel Milk Protein Ingredients for Use in Infant Formulas: Research on Biological Functions. Trends Food Sci. Technol..

[78] Kurzbach D., Platzer G., Schwarz T.C., Henen M.A., Konrat R., Hinderberger D. (2013). Cooperative Unfolding of Compact Conformations of the Intrinsically Disordered Protein Osteopontin. Biochemistry.

[79] da Silva A.P.B., Ellen R.P., Sørensen E.S., Goldberg H.A., Zohar R., Sodek J. (2009). Osteopontin Attenuation of Dextran Sulfate Sodium-Induced Colitis in Mice. Lab. Investig..

[80] Liu L., Jiang R., Lönnerdal B. (2019). Assessment of Bioactivities of the Human Milk Lactoferrin-Osteopontin Complex in Vitro. J. Nutr. Biochem..

[81] Liu L., Jiang R., Liu J., Lönnerdal B. (2020). The Bovine Lactoferrin-Osteopontin Complex Increases Proliferation of Human Intestinal Epithelial Cells by Activating the PI3K/Akt Signaling Pathway. Food Chem..

[82] Dall’Asta C., Florio P., Lammardo A.M., Prandi B., Mazzeo T., Budelli A., Pellegrini N. (2015). Development of an in Vitro Digestive Model for Studying the Peptide Profile of Breast Milk. Int. J. Food Sci. Nutr..

[83] Wada Y., Lönnerdal B. (2015). Bioactive Peptides Released from in Vitro Digestion of Human Milk with or without Pasteurization. Pediatr. Res..

[84] Rittling S.R., Wejse P.L., Yagiz K., Warot G.A., Hui T. (2014). Suppression of Tumour Growth by Orally Administered Osteopontin Is Accompanied by Alterations in Tumour Blood Vessels. Br. J. Cancer.

[85] Christensen B., Nielsen N.R., Sørensen M.R., Jacobsen L.N., Ostenfeld M.S., Sørensen E.S. (2023). Naturally Occurring *N*-Terminal Fragments of Bovine Milk Osteopontin Are Transported across Models of the Intestinal Barrier. Biomedicines.

[86] Yokosaki Y., Tanaka K., Higashikawa F., Yamashita K., Eboshida A. (2005). Distinct Structural Requirements for Binding of the Integrins Avβ6, Avβ3, Avβ5, A5β1 and A9β1 to Osteopontin. Matrix Biol..

[87] Kläning E., Christensen B., Bajic G., Hoffmann S.V., Jones N.C., Callesen M.M., Andersen G.R., Sørensen E.S., Vorup-Jensen T. (2015). Multiple Low-Affinity Interactions Support Binding of Human Osteopontin to Integrin AXβ2. Biochim. Biophys. Acta.

[88] Ashkar S., Weber G.F., Panoutsakopoulou V., Sanchirico M.E., Jansson M., Zawaideh S., Rittling S.R., Denhardt D.T., Glimcher M.J., Cantor H. (2000). Eta-1 (Osteopontin): An Early Component of Type-1 (Cell-Mediated) Immunity. Science.

[89] Ito K., Kon S., Nakayama Y., Kurotaki D., Saito Y., Kanayama M., Kimura C., Diao H., Morimoto J., Matsui Y. (2009). The Differential Amino Acid Requirement within Osteopontin in A4 and A9 Integrin-Mediated Cell Binding and Migration. Matrix Biol..

[90] Gassler N., Autschbach F., Gauer S., Bohn J., Sido B., Otto H.F., Geiger H., Obermüller N. (2002). Expression of Osteopontin (Eta-1) in Crohn Disease of the Terminal Ileum. Scand. J. Gastroenterol..

[91] Agnholt J., Kelsen J., Schack L., Hvas C.L., Dahlerup J.F., Sørensen E.S. (2007). Osteopontin, a Protein with Cytokine-like Properties, Is Associated with Inflammation in Crohn’s Disease. Scand. J. Immunol..

[92] Da Silva A.P.B., Pollett A., Rittling S.R., Denhardt D.T., Sodek J., Zohar R. (2006). Exacerbated Tissue Destruction in DSS-Induced Acute Colitis of OPN-Null Mice Is Associated with Downregulation of TNF-Alpha Expression and Non-Programmed Cell Death. J. Cell. Physiol..

[93] Kanwar J.R., Kanwar R.K., Stathopoulos S., Haggarty N.W., MacGibbon A.K.H., Palmano K.P., Roy K., Rowan A., Krissansen G.W. (2016). Comparative Activities of Milk Components in Reversing Chronic Colitis. J. Dairy Sci..

[94] Ge X., Lu Y., Leung T.-M., Sørensen E.S., Nieto N. (2013). Milk Osteopontin, a Nutritional Approach to Prevent Alcohol-Induced Liver Injury. Am. J. Physiol.-Gastrointest. Liver Physiol..

[95] Ren S., Hui Y., Goericke-Pesch S., Pankratova S., Kot W., Pan X., Thymann T., Sangild P.T., Nguyen D.N. (2019). Gut and Immune Effects of Bioactive Milk Factors in Preterm Pigs Exposed to Prenatal Inflammation. Am. J. Physiol.-Gastrointest. Liver Physiol..

[96] Aasmul-Olsen K., Henriksen N.L., Nguyen D.N., Heckmann A.B., Thymann T., Sangild P.T., Bering S.B. (2021). Milk Osteopontin for Gut, Immunity and Brain Development in Preterm Pigs. Nutrients.

[97] Jiang R., Lönnerdal B. (2020). Evaluation of Bioactivities of Bovine Milk Osteopontin Using a Knockout Mouse Model. J. Pediatr. Gastroenterol. Nutr..

[98] Donovan S.M., Monaco M.H., Drnevich J., Kvistgaard A.S., Hernell O., Lönnerdal B. (2014). Bovine Osteopontin Modifies the Intestinal Transcriptome of Formula-Fed Infant Rhesus Monkeys to Be More Similar to Those That Were Breastfed. J. Nutr..

[99] Christensen B., Buitenhuis A.J., Jacobsen L.N., Ostenfeld M.S., Sørensen E.S. (2023). The Effect of Human and Bovine Milk Osteopontin on Intestinal Caco-2 Cells: A Transcriptome Comparison. Nutrients.

[100] Zhao G., Shi L., Qiu D., Hu H., Kao P.N. (2005). NF45/ILF2 Tissue Expression, Promoter Analysis, and Interleukin-2 Transactivating Function. Exp. Cell Res..

[101] Malek T.R., Castro I. (2010). Interleukin-2 Receptor Signaling: At the Interface between Tolerance and Immunity. Immunity.

[102] Lönnerdal B., Kvistgaard A.S., Peerson J.M., Donovan S.M., Peng Y. (2016). Growth, Nutrition, and Cytokine Response of Breast-Fed Infants and Infants Fed Formula With Added Bovine Osteopontin. J. Pediatr. Gastroenterol. Nutr..

[103] Jiang R., Lönnerdal B. (2014). Transcriptomic Profiling of Intestinal Epithelial Cells in Response to Human, Bovine and Commercial Bovine Lactoferrins. BioMetals.

[104] Toyonaga T., Nakase H., Ueno S., Matsuura M., Yoshino T., Honzawa Y., Itou A., Namba K., Minami N., Yamada S. (2015). Osteopontin Deficiency Accelerates Spontaneous Colitis in Mice with Disrupted Gut Microbiota and Macrophage Phagocytic Activity. PLoS ONE.

[105] Atarashi K., Tanoue T., Oshima K., Suda W., Nagano Y., Nishikawa H., Fukuda S., Saito T., Narushima S., Hase K. (2013). Treg Induction by a Rationally Selected Mixture of Clostridia Strains from the Human Microbiota. Nature.

[106] Aggarwal N., Deerhake M.E., DiPalma D., Shahi S.K., Gaggioli M.R., Mangalam A.K., Shinohara M.L. (2021). Secreted Osteopontin from CD4+ T Cells Limits Acute Graft-versus-Host Disease. Cell Rep..

[107] Ge X., Leung T.-M., Arriazu E., Lu Y., Urtasun R., Christensen B., Fiel M.I., Mochida S., Sørensen E.S., Nieto N. (2014). Osteopontin Binding to Lipopolysaccharide Lowers Tumor Necrosis Factor-α and Prevents Early Alcohol-Induced Liver Injury in Mice. Hepatology.

[108] Das S., Song Z., Han H., Ge X., Desert R., Athavale D., Babu Komakula S.S., Magdaleno F., Chen W., Lantvit D. (2022). Intestinal Osteopontin Protects From Alcohol-Induced Liver Injury by Preserving the Gut Microbiome and the Intestinal Barrier Function. Cell. Mol. Gastroenterol. Hepatol..

[109] Chen J., Zeng P., Gong L., Zhang X., Ling Z., Bi K., Shi F., Wang K., Zhang Q., Jiang J. (2022). Osteopontin Exacerbates High-Fat Diet-Induced Metabolic Disorders in a Microbiome-Dependent Manner. mBio.

[110] Smith B.N., Hannas M., Orso C., Martins S.M.M.K., Wang M., Donovan S.M., Dilger R.N. (2020). Dietary Osteopontin-Enriched Algal Protein as Nutritional Support in Weaned Pigs Infected with F18-Fimbriated Enterotoxigenic Escherichia Coli. J. Anim. Sci..

[111] Lin E.Y.-H., Xi W., Aggarwal N., Shinohara M.L. (2023). Osteopontin (OPN)/SPP1: From Its Biochemistry to Biological Functions in the Innate Immune System and the Central Nervous System (CNS). Int. Immunol..

[112] Golińska E., Strus M., Tomusiak-Plebanek A., Więcek G., Kozień Ł., Lauterbach R., Pawlik D., Rzepecka-Węglarz B., Kędzierska J., Dorycka M. (2020). Coagulase-Negative Staphylococci Contained in Gut Microbiota as a Primary Source of Sepsis in Low- and Very Low Birth Weight Neonates. J. Clin. Med..

[113] West C.E., Kvistgaard A.S., Peerson J.M., Donovan S.M., Peng Y.-M., Lönnerdal B. (2017). Effects of Osteopontin-Enriched Formula on Lymphocyte Subsets in the First 6 Months of Life: A Randomized Controlled Trial. Pediatr. Res..

[114] Choi J.-S., Cha J.-H., Park H.-J., Chung J.-W., Chun M.-H., Lee M.-Y. (2004). Transient Expression of Osteopontin MRNA and Protein in Amoeboid Microglia in Developing Rat Brain. Exp. Brain Res..

[115] Lee M.Y., Choi J.S., Lim S.W., Cha J.H., Chun M.H., Chung J.W. (2001). Expression of Osteopontin MRNA in Developing Rat Brainstem and Cerebellum. Cell Tissue Res..

[116] Selvaraju R., Bernasconi L., Losberger C., Graber P., Kadi L., Avellana-Adalid V., Picard-Riera N., Baron Van Evercooren A., Cirillo R., Kosco-Vilbois M. (2004). Osteopontin Is Upregulated during in Vivo Demyelination and Remyelination and Enhances Myelin Formation in Vitro. Mol. Cell. Neurosci..

[117] Chen W., Ma Q., Suzuki H., Hartman R., Tang J., Zhang J.H. (2011). Osteopontin Reduced Hypoxia-Ischemia Neonatal Brain Injury by Suppression of Apoptosis in a Rat Pup Model. Stroke.

[118] Horta B.L., Loret de Mola C., Victora C.G. (2015). Breastfeeding and Intelligence: A Systematic Review and Meta-Analysis. Acta Paediatr..

[119] Xie Q., Zhang Y., Zhang J., Cui D., Zhou Q., Guo M. (2022). Promotion Effect of the Blend Containing 2′-FL, OPN and DHA on Oligodendrocyte Progenitor Cells Myelination in Vitro. Front. Nutr..

[120] Joung S., Fil J.E., Heckmann A.B., Kvistgaard A.S., Dilger R.N. (2020). Early-Life Supplementation of Bovine Milk Osteopontin Supports Neurodevelopment and Influences Exploratory Behavior. Nutrients.

[121] Yamniuk A.P., Burling H., Vogel H.J. (2009). Thermodynamic Characterization of the Interactions between the Immunoregulatory Proteins Osteopontin and Lactoferrin. Mol. Immunol..

[122] Jiang R., Liu L., Du X., Lönnerdal B. (2020). Evaluation of Bioactivities of the Bovine Milk Lactoferrin-Osteopontin Complex in Infant Formulas. J. Agric. Food Chem..

[123] MacNeil R.L., Berry J., D’Errico J., Strayhorn C., Piotrowski B., Somerman M.J. (1995). Role of Two Mineral-Associated Adhesion Molecules, Osteopontin and Bone Sialoprotein, during Cementogenesis. Connect. Tissue Res..

[124] McKee M.D., Nanci A. (1996). Osteopontin at Mineralized Tissue Interfaces in Bone, Teeth, and Osseointegrated Implants: Ultrastructural Distribution and Implications for Mineralized Tissue Formation, Turnover, and Repair. Microsc. Res. Tech..

[125] Nurrohman H., Carter L., Barnes N., Zehra S., Singh V., Tao J., Marshall S.J., Marshall G.W. (2022). The Role of Process-Directing Agents on Enamel Lesion Remineralization: Fluoride Boosters. Biomimetics.

[126] Burling H., Sørensen E.S., Bertelsen H., Jørgensen A.S., Graverholt G. (2005). Use of Osteopontin in Dental Formulations. U.S. Patent.

[127] Kristensen M.F., Zeng G., Neu T.R., Meyer R.L., Baelum V., Schlafer S. (2017). Osteopontin Adsorption to Gram-Positive Cells Reduces Adhesion Forces and Attachment to Surfaces under Flow. J. Oral Microbiol..

[128] Schlafer S., Meyer R.L., Sutherland D.S., Städler B. (2012). Effect of Osteopontin on the Initial Adhesion of Dental Bacteria. J. Nat. Prod..

[129] Schlafer S., Raarup M.K., Wejse P.L., Nyvad B., Städler B.M., Sutherland D.S., Birkedal H., Meyer R.L. (2012). Osteopontin Reduces Biofilm Formation in a Multi-Species Model of Dental Biofilm. PLoS ONE.

[130] Kristensen M.F., Sørensen E.S., Del Rey Y.C., Schlafer S. (2022). Prevention of Initial Bacterial Attachment by Osteopontin and Other Bioactive Milk Proteins. Biomedicines.

